# Melting Temperature Depression of Polymer Single Crystals: Application to the Eco-Design of Tie-Layers in Polyolefinic-Based Multilayered Films

**DOI:** 10.3390/polym14081622

**Published:** 2022-04-17

**Authors:** Juan F. Vega, Virginia Souza-Egipsy, M. Teresa Expósito, Javier Ramos

**Affiliations:** 1 BIOPHYM, Departamento de Física Macromolecular, Instituto de Estructura de la Materia, IEM-CSIC, c/Serrano 113 bis, 28006 Madrid, Spain; virginia.souza-egipsy@csic.es (V.S.-E.); j.ramos@csic.es (J.R.); 2 Interdisciplinary Platform for Sustainable Plastics towards a Circular Economy, SUSPLAST-CSIC, 28006 Madrid, Spain; 3 ESCET, Universidad Rey Juan Carlos, c/Tulipán, 28933 Madrid, Spain; teresa.exposito@urjc.es

**Keywords:** single crystals, melting temperature depression, compatibility, eco-design, multilayers, mechanical recycling

## Abstract

In this paper, we describe a method for determining polymer compatibility, which will aid in establishing the requirements of polyolefinic materials for the eco-design of multilayer films for mechanical recycling while avoiding the use of reactive tie layers. Our ultimate goal is to define the molecular characteristics of the polyolefinic structural layer that improve compatibility with the tie layer during mechanical recycling. We have investigated the melting temperature depression of single crystals of various polyethylenes embedded in commercial polymeric matrices with various functionalities (ester, acrylate, acetate and methacrylic acid sodium ionomer), which can be potentially used as tie layers. We demonstrate how the concentration and molecular architecture of the matrices affect the melting temperature of the embedded single crystals differently depending on the latter’s molecular architecture. The main finding indicates that the tie layers are more compatible with linear polyethylene than with branched polyethylenes. Indeed, our results show that the heterogeneous Ziegler–Natta linear low-density polyethylene is incompatible with all of the tie layers tested. The depression of melting temperatures observed are in excellent agreement with the results obtained by investigating the rheological behaviour and morphological features of solution-mixed blends in which segmental interactions between polymeric chains have been, in theory, maximized. Because Ziegler–Natta linear density polyethylene is one of the most commonly used polymers as a structural layer in multi-layer applications, the findings of this study are useful as they clearly show the unsuitability of this type of polyethylene for recycling from an eco-design standpoint. The specific molecular requirements for polyethylene layers (branching content less than 0.5/100 carbon atoms) can be specified for use in packaging, guiding the eco-design and valorisation of recycled multi-layered films containing this material.

## 1. Introduction

Global plastics production almost reached 370 million tonnes in 2020, from those 58 million were produced in Europe [1,2,3,4]. The packaging industry is the most important market for this massive amount of plastic, accounting for roughly 40% of total demand [5]. In this specific case, the main concern about recyclability is focussed on multi-layered films which are widely used in a variety of applications including food [6], innovative medicine and pharmacy [7,8,9], biotechnology [10], and electronics and dielectrics [11,12,13]. Polyethylene (PE), particularly linear low-density polyethylene (LLDPE), is one of the most used polymers for these applications due to its superior physical and mechanical properties. Low-density polyethylene (LDPE) is also used, but to a lesser extent. In multilayer packaging, these materials are combined with polar polymers such as polyamides (PA), ethylene/vinyl alcohol copolymers (EVOH) and/or polyethylene terephthalate (PET) [14,15,16,17]. Other components of the multilayers include ethylene copolymers functionalized with anhydride and acid groups, which serve to promote adhesion between olefinic and polar layers [18,19]. During the co-extrusion process, these groups react covalently with the polar layer. This fact limits the possibility of mechanical recycling of multilayers because the reaction may produce undesirable gels and may also impede layer separation if a mechanical/chemical recycling approach is desired. To avoid these issues, we propose that non-covalent interactions among the components are more appropriate, but also challenging.

We have focused our attention in the study on the interactions between different polyolefinic layers and ethylene copolymers with ethyl acrylate (EEA), acetate (EVA), and ionomers of acrylic acid (EMAA). Due to its chemical nature, a polycaprolactone homopolymer (PCL) with a monomeric unit containing a long aliphatic -CH_2_- segment was also included as a possible candidate. Comprehensive reviews on the effect of molecular architecture of the different PE types on compatibility can be found elsewhere [20,21]. The studies focused on blends of PE with EEA copolymers are quite limited [22,23,24]. Conversely, the study of PE blends with EVA [25,26,27,28,29,30] and EMAA [31,32,33] has generated an interesting number of publications. In general, incompatibility is reported, but it is not easy to find systematic studies about the effect of the molecular architecture of the polymers in the compatibility. Investigations dealing with blends based on PE and PCL are also scarce, but may be of interest because the chemical structures of both polymers are fairly analogous [34].

In this paper we have measured the melting point depression to investigate the interactions between the polymeric components of a mixture, at least one of which is semicrystalline [35]. It is widely recognized that the presence of a second component in polymeric blends can change the characteristic crystalline structure of the semicrystalline polymer. The use of single crystals of this semicrystalline polymer embedded in a polymeric matrix of the other component solves this problem by ensuring that the crystalline structural parameters remain constant with composition. Under these conditions the relationship between melting temperature variation and mixing thermodynamics is straightforward. This methodology was successfully applied to linear PE single crystals embedded in paraffins [36]. A strong depression is caused by pure entropic interactions between the mixture’s components in this case. Using this method, the compatibility of HDPE and LDPE was also investigated [37]. In this case it was difficult to properly separate the enthalpic and entropic contributions due to branches, expected to go in opposite directions. Notwithstanding, the melting temperature depression allowed the extraction of a critical value of the branching content in LDPE for the compatibility with HDPE of around 2%. We present now the melting temperature depression data of different PE single crystals immersed in different matrices used in applications as tie layers. We demonstrate how the matrices’ concentration and molecular structure affect crystal melting, and present interesting information about the effect of molecular architecture on compatibility and recyclability chances in the melt. The results obtained were tested in solution-mixed blends, where segmental interaction between polymeric chains was, in theory, maximized. The results obtained in this work show the great potential of the technique for studying interactions between dissimilar polymers, as well as for defining the implications of these interactions for compatibility and recyclability.

## 2. Materials and Methods

### 2.1. Materials

Four PE grades, one linear and three short chain branched, have been used for the present study. The linear and two of the branched samples are non-commercial single-site catalysed PEs, designated as PExx, where xx indicates the branching index as the number of branches per 100 carbon atoms. An additional commercial Ziegler–Natta LLDPE purchased from Sigma Aldrich/Merck, designated as PE_ZN was also used. Molecular (branching content every 100 carbon atoms and molecular weight distribution) and physical properties (melt flow index, density and melting temperature of the pellets, as received) of these samples are collected in Table 1. More details on PE characterization can be found elsewhere [38].

The materials used as a second component of the blends are listed in Table 2, together with their molecular and physical properties. PCL homopolymer, and EEA and EVA copolymers have been purchased from Sigma Aldrich/Merck. EMAANa copolymer of tradename Surlyn PC2000 has been kindly donated by Dow Chemical Ibérica (Spain). All these copolymers are typical tie layers used in packaging manufacture [39].

### 2.2. Sample Preparation

Fragmented pellets of EEA (10 mg·mL^−1^), EVA (10 mg·mL^−1^) and EMAANa (5 mg·mL^−1^) were dissolved in 50 mL p-xylene (EEA and EVA) and 1-pentanol (EMAANa) by heating up the solution to 100 °C Cover 2–3 h. A fine dispersion of the samples was obtained by simply cooling to room temperature. Dissolution of PCL (5 mg·mL^−1^) was done in acetone. Solutions of polyethylene in p-xylene were prepared with a concentration of 5 mg·mL^−1^ (PE0.0) and 2.5 mg·mL^−1^ (PE0.3, PE0.5 and PE_ZN). A self-seeding procedure was employed before the samples were isothermally crystallized from solution. Solutions were heated to 130 °C and cooled within a few minutes to a temperature T_ci_ (see Table 3), where a fast crystallization took place in a short period of time (15–20 min). Then the suspensions were slowly heated (10 °C per hour) to a self-seeding temperature, T_s_, and kept at that temperature for 15 min. The solutions were then recrystallized to obtain single crystals at T_c_ over 24 h. Blends were done by adding 15, 25, 50, or 75% of each dispersion (PCL, EEA, EVA 5 mg·mL^−1^ in p-xylene and EMAANa 5 mg·mL^−1^ in 1-pentanol) to the single crystal dispersions and kept under agitation at 65 °C until solvent evaporation. Samples were dried under vacuum for 48 h before further analysis.

For selected cases, binary solution-mixed blends of PE and EVA were prepared using the samples described in Table 1 and Table 2. Samples containing 80% of PE and 20% of EVA were prepared by dissolving the polymer pellets in hot p-xylene followed by precipitation by solvent evaporation. The filtered materials were washed with acetone and finally dried in a vacuum oven over 24 h to ensure complete removal of the liquids. Films (500 μm thick) of resulting blends were prepared in a hot-press at 160 °C for 5 min for rheological measurements. 

### 2.3. Morphology of the Single Crystals

The single crystals dispersed in the p-xylene solutions were deposited onto carbon-coated grids for their examination by transmission electron microscopy (TEM). A JEM-2100 (JEOL Ltd., Tokyo, Japan) microscope operated at 200 kV equipped with a CCD camera ORIUS SC1000 Model 832 (GATAN) was used.

### 2.4. Thermal Properties

A differential scanning calorimeter DSC-7 (Perkin Elmer Inc., Waltham, MA, USA) under a ultra-high purity nitrogen atmosphere and calibrated with Indium was used. Samples of approximately 2 mg were encapsulated in aluminium pans and sealed. Samples were heated from 20 °C to 160 °C at 10 °C·min^−1^. Cooling and subsequent heating scans were also registered but not analysed at this stage. The contribution from an empty pan was subtracted from each experiment. The fraction of crystals, α, was obtained from the measured melting enthalpy considering ΔH^0^_m_ = 293 J·g^−1^ as the equilibrium melting enthalpy of fully crystalline PE [42], and ΔH^0^_m_ = 139.5 J·g^−1^ for fully crystalline PCL [43].

### 2.5. Rheological Testing

Small amplitude strain sweeps and frequency sweeps were carried out in order to locate the linear viscoelastic region and to determine the basic linear viscoelastic properties. The experiments were performed in a stress-controlled Bohlin CVO rheometer (Malvern Instruments, Worcestershire, UK) in dynamic mode. Dynamic oscillatory shear measurements were performed at a temperature of 150 °C within the frequency range 0.01–100 rad·s^−1^. The samples were allowed to equilibrate for at least 5 min at each temperature. The applied shear stress amplitudes corresponded to a shear strain well below 0.1, which was proven to be in the linear viscoelastic regime for the neat materials and the blends. The properties measured were the storage and the loss moduli, G′(ω) and G″(ω), as well as the out-of-phase component of the complex viscosity, η″(ω).

Different models were applied in order to explain the rheological properties of the blends. The Palierne model allows one to describe the linear response in immiscible systems in terms of the properties of each phase, the matrix (m) and the dispersed phase (d) [44]:

(1) $$G^{*}\left(\omega \right){=G}_{m}^{*}\left(\omega \right)\frac{1+3\sum_{i}\phi_{i}H_{i}\left(\omega \right)}{1-2\sum_{i}\phi_{i}H_{i}\left(\omega \right)}$$

where H_i_(ω) is given by:

(2) $$H_{i}\left(\omega \right)=\frac{4\left(\frac{\alpha }{R}\right)\left({2G}_{m}^{*}{+5G}_{d}^{*}\right)+\left(G_{d}^{*}{-G}_{m}^{*}\right)\left({16G}_{m}^{*}{+19G}_{d}^{*}\right)}{4o\left(\frac{\alpha }{R}\right)\left(G_{m}^{*}{+G}_{d}^{*}\right)+\left({2G}_{d}^{*}{+3G}_{m}^{*}\right)\left({16G}_{m}^{*}{+19G}_{d}^{*}\right)}$$

The interfacial tension, α, the dispersed droplets average radius, R, and the volume fraction of the dispersed phase, ϕ, are the parameters that modulate the enhancement of the viscoelastic properties in the immiscible blend with respect to that of the pure components. For miscible blends, Groves et al. found agreement between rheological measurements and predictions derived from the double reptation theory, by empirically varying the exponent C of the generalized blending law [45]:

(3) $$G\left(t\right)={\left[\underset{i}{\sum }\phi_{i}G_{i}^{1/C}\right]}^{C}\;$$

where ϕ_i_ is the blend component volume fraction, G_i_ is the relaxation modulus of each component, and C is an empirical parameter, mainly dependent on the Newtonian viscosity of each pure component, η_0_. If the pure components show similar values of η_0_, the value of C is close to 2. The reader is referred to the work by Lee and Denn for detailed information [46]. The corresponding values of G′(ω) and G″(ω) can be obtained from the calculated G(t) values in Equation (3), as the Fourier sine and cosine transforms of G(t), respectively.

### 2.6. Atomic Force Microscopy

Atomic force microscopy (AFM) imaging of the films used for rheological testing was carried out using a µTA™ 2990 Micro-Thermal Analyzer (TA Instruments, Inc., New Castle, DE, USA). Topography micrographs were recorded in contact mode at room temperature. To this end, a V-shaped silicon nitride probe with a cantilever length of 200 µm and a spring constant of 0.032 N m^−1^ was used. The dimension of the images was variable between 20 and 5 µm^2^. The blends were compression-moulded at 160 °C. During compression the blends were sandwiched between Mylar sheets, heated at 160 °C under minimal pressure for 5 min, and quenched at room temperature. The polymeric films have been supported on glass wafers for morphological observations. The images were analysed with the Gwyddion software [47]. 

## 3. Results and Discussion

### 3.1. Characterization of the Samples

**Figure 1:**
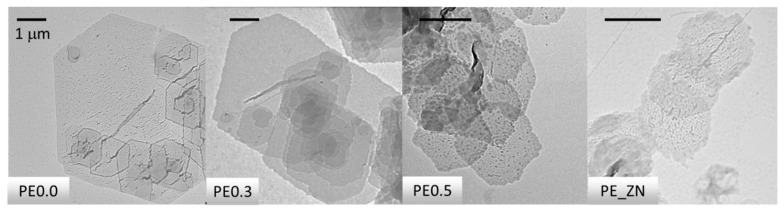
TEM images of the single crystals in PE00, PE0.3, PE0.5 and PE_ZN samples. The bars correspond to a size scale of 1 μm in all cases.

Figure 1 depicts the TEM results for the single crystals of the PE samples studied. The images show that the crystals adsorbed onto the carbon coated grids are uniform, indicating that they have self-nucleated. As previously reported, multilayer spiral growth is observed, particularly in the case of PE0.0 and PE0.3 samples [48]. Spiral growth has been observed frequently in lamellar single crystals, especially for n-paraffins and polyethylene precipitated from dilute xylene solution. The origin of these structures has been attributed to screw dislocations caused by the interference of two growing single crystals, as well as the pyramidal nature of the single crystals [17,49,50]. It is noteworthy that all samples were able to generate single crystals from dilute solution, including the commercial polydisperse PE-ZN. Slightly branched samples PE0.3 and PE0.5 generate single crystals that closely resemble the truncated lozenge shape of the linear PE0.0, but with slightly curved and serrated edges. The single crystals of highly branched commercial PE ZN have a rounded habit, though the lozenge shape can be guessed. As the amount of SCB in the polymer increases, the size of the crystals decreases. Furthermore, as the SCB content increases, the morphology of the single crystal deteriorates. This observation is consistent with Keller’s hypotheses, which directly linked crystal morphology to fold structure: the longer the loops, the poorer the morphology [51,52].

**Figure 2:**
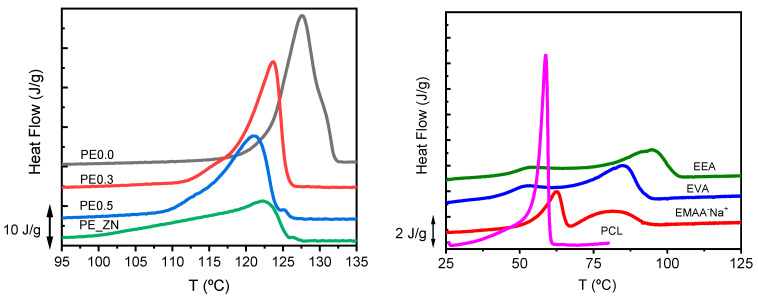
DSC traces of the single crystals of the polyethylenes (**left**) and matrices (**right**) at a heating rate of 10 °C·min^−1^.

Figure 2 compares the DSC scans recorded for the single crystal mats of the PE samples studied (PE00, PE0.3, PE0.5 and PE_ZN) at a scan rate of 10 °C·min^−1^. As repeatedly reported in the literature for the linear PE single crystals, the heating curves show crystal thickening during heating [53,54,55,56]. For PE0.0 single crystals, the main peak at 127.6 °C represents the melting of the original crystals, while the upper shoulder, around 130 °C, corresponds to the melting of recrystallized material (annealing process). This crystal thickening phenomenon is not observed in single crystals obtained from branched PE samples, as previously reported both experimentally and through computer simulations [48,57]. The melting peaks in PE0.3 and PE0.5 samples are well defined, which corresponds to homogeneous populations of crystals of 12 to 9 nm of lateral thickness [48]. The melting trace of PE_ZN single crystals is definitively broad. This is likely due to a more heterogeneous population of crystals as a consequence of the heterogeneous distribution of comonomer in the polymeric chains of this type of materials. Table 3 shows the thermal and microstructural properties of the single crystals derived from DSC experiments, melting temperature, T_m_; melting enthalpy, ΔH_m_; and crystalline fraction, α. The expected decreasing trends in all thermal properties as comonomer content increases is observed.

The thermal properties of the homo and copolymers used as matrices have been also obtained. The DSC traces of PCL, EEA, EVA and EMAANa samples are presented in Figure 2 (right). The melting point of all these samples is well below that of the PE single crystals studied previously. Also, crystallinity values are lower than those measured in PE single crystals (see Table 2). These are significant facts because, in systems composed of single crystals embedded in matrices, the melting of the former will occur in a completely amorphous environment. Thus, the eventual changes in PE single crystals thermodynamics will be due to the specific interactions with the amorphous matrix at a given composition, rather than to the microstructural features of the crystals as they remain unaltered.

### 3.2. Melting Temperature Depression Analysis

**Figure 3:**
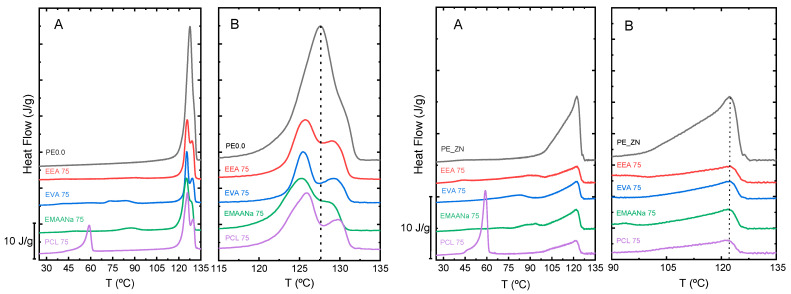
DSC melting traces of PE0.0 (**left**) and PE_ZN (**right**) single crystals mixtures (25/75) with the different branched matrices: (**A**) whole DSC traces and (**B**) detail of PE single crystals melting zone. The dashed line indicates the melting temperature of the corresponding single crystals.

In Figure 3A the experimental melting endotherms at a heating rate of 10 °C·min^−1^ obtained for the PE0.0 (left) and PE_ZN (right) single crystals mixtures with 75% of the second component (EEA, EVA, EMAANa and PCL) are displayed. In both cases two well separated main peaks are observed, the low-temperature peaks that correspond to the melting of the second component (matrix), and the high-temperature peaks that are associated with the melting of the single crystals.

Figure 3B show details of the high-temperature peaks corresponding to the melting of single crystals surrounded by melted amorphous matrices for each of the PE0.0 (left) and PE_ZN (right) mixtures. A clear shift to lower melting temperatures is observed for all the mixtures in the case of PE0.0 single crystals. Regardless, the shift is absent when the single crystals are from the branched PE_ZN sample. It is interesting to note that the thickening process in the linear PE0.0 single crystals is still more visible in the blends. Moreover, this process seems to be quite similar irrespective of the matrix component.

**Figure 4:**
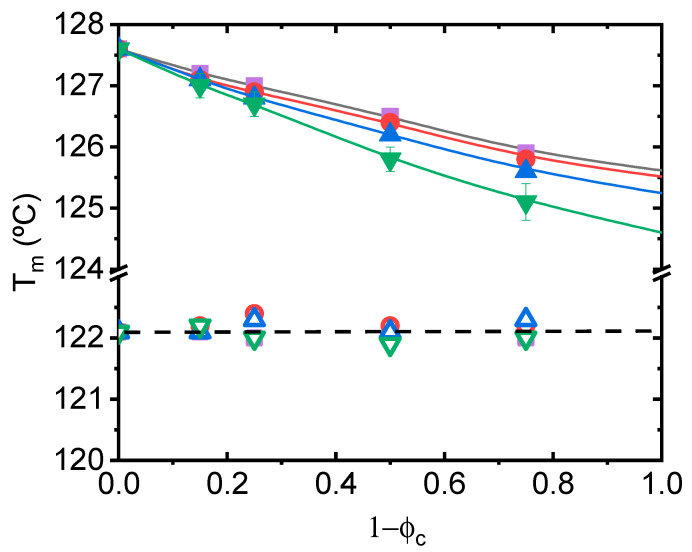
Melting peak temperature compositional dependence for PE0.0 (solid symbols) and PE_ZN (hollow symbols) mixed with EEA (circles), EVA (up triangles), EMAANa (down triangles) and PCL (squares).

For the evaluation of the melting point depression, the melting peaks (T_m_) have been located and plotted as a function of composition (fraction of second component) in Figure 4, for the whole set of mixtures studied. We find that T_m_ decreases with increasing concentration of PCL, EEA, EVA and EMAANa in the case of PE0.0 single crystals. The level of the experimental depression is more conspicuous in the EMAANa matrix (ΔT = 2.5 °C) than in the PCL matrix (ΔT = 1.5 °C). The level of depression in melting temperature of PE0.0 single crystals closely resembles that found in HDPE single crystals embedded in LLDPEs, for which values up to ΔT = 1.6 °C were determined [37]. The addition of these components to PE_ZN single crystals does not cause any effect. These results suggest the existence of a clear effect of the molecular architecture of the PE component in the compatibility with the different matrices, as PE0.0 and PE_ZN samples represent two extreme cases. It should be noted that PE blends with EEA are rare in the literature. On the contrary, as stated in the Introduction section, PE/EVA [25,26,27,28,29,30] and PE/EMAA [31,32,33] blends have been extensively studied, and in general, incompatibility has been reported. It should be noted that the majority of the studies were conducted for blends of LDPE or mixed PE types and with copolymers with higher comonomer contents than those used by us. As a result, obtaining information from these works about the effect of specific molecular features in compatibility is difficult. 

**Figure 5:**
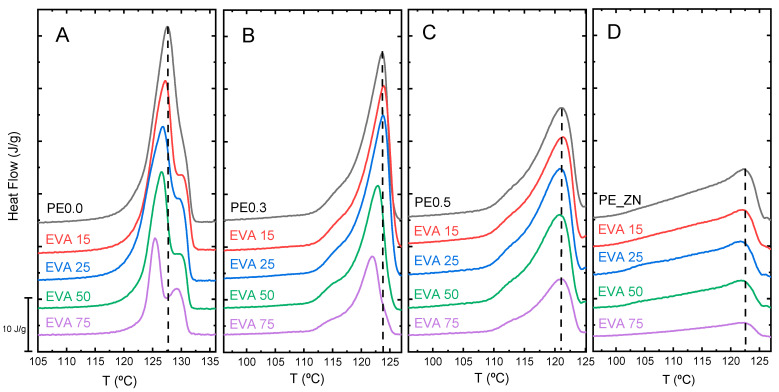
DSC melting traces of PE0.0 (**A**), PE0.3 (**B**), PE0.5 (**C**) and PE_ZN (**D**) single crystals mixtures with EVA in the whole compositional range.

In order to systematically explore the effect of the molecular architecture, we selected the mixtures of the single crystals of the whole set of PE samples in Table 1, with one of the matrices, the EVA copolymer. Figure 5 depicts the DSC traces for the single crystal types PE0.0, PE0.3, PE0.5, and PE ZN with the EVA matrix over the entire compositional range. In Figure 5A, we clearly see: (i) a noticeable decrease in melting temperature, dependent on composition, and (ii) a thickening of the single crystals, a relevant phenomenon typically observed on heating for thin polymeric lamellae of linear PE. This crystal thickening process seems to be more pronounced in the mixtures with highest content in the EVA copolymer matrix. A close inspection of the DSC traces obtained in the high-temperature portion for these compositions already indicates a similar melting trace of the PE0.0 single crystals, irrespective of the matrix counterpart in the mixture, as it was observed in Figure 3A. The PE0.3/EVA mixtures in Figure 5B also display melting temperature depression, as PE0.0/EVA mixtures, but to a lesser extent. In contrast, in the case of PE0.5/EVA and PE_ZN/EVA mixtures in Figure 5C,D, the compositional variation neither causes any noticeable change in the melting temperature location nor in the shape of melting peak.

**Figure 6:**
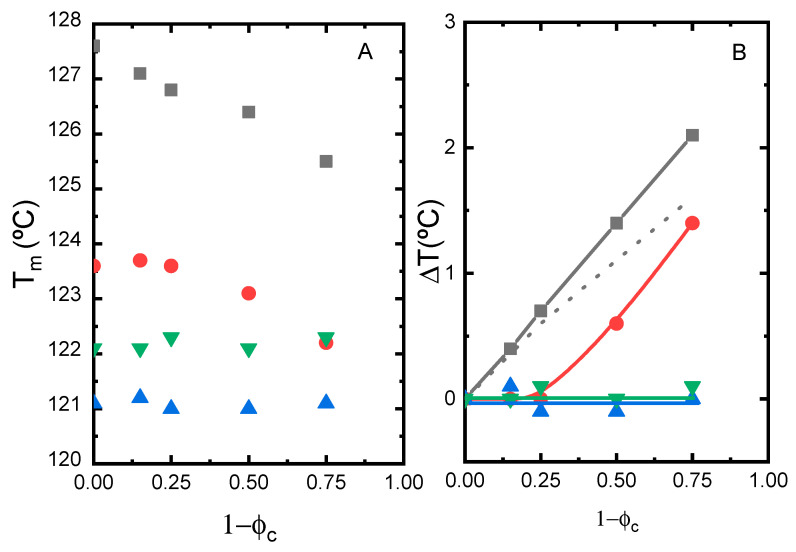
(**A**) Compositional dependence of the melting temperature of the single crystals embedded in EVA matrix. (**B**) Melting temperature depression of the mixtures studied: PE0.0 (squares), PE0.3 (circles), PE0.5 (down triangles) and PE_ZN (up triangles). The dotted line indicates the behaviour of HDPE single crystals embedded in LDPE from the literature [29].

We clearly find that T_m_ decreases with increasing concentration of EVA in the case of PE0.0 and PE0.3 single crystals, as observed in Figure 6. The level of the experimental depression is more pronounced in PE0.0 single crystals (ΔT = 2.1 °C), it is still present in PE03 single crystals (ΔT = 1.4 °C), but completely vanishes in PE single crystals with branching content higher than 0.3 per 100 carbon atoms. The result suggests that PE samples with branching content higher than 0.3 per 100 carbon atoms are likely incompatible with customary tie layers used for the manufacture of packaging films such as EVA copolymers. This result has negative implications for the mechanical recycling of polyolefinic materials so widely used in the fabrication of multilayer films such as Ziegler–Natta LLDPEs, characterized by their heterogeneity and relatively high comonomer content.

### 3.3. Rheological Properties of Selected Blends

**Figure 7:**
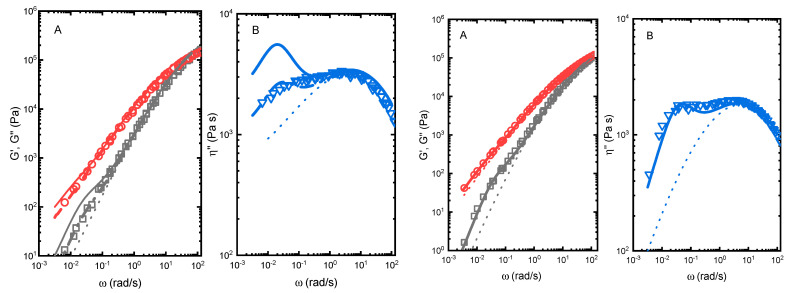
(**A**) Storage (□) and loss (Ο) moduli and (**B**) out-of-phase component of complex viscosity (σ) versus angular frequency for PE0.0/EVA 80/20 blend (**left**) and PE_ZN/EVA 80/20 (**right**) at T = 150 °C. The dotted curves are the calculations for a miscible blend. The solid lines represent the results of the Palierne model for immiscible blends with α/R = 0.8 kN·m^−2^. The dashed lines represent the behaviour obtained for a partially miscible blend with X = 0.6 and α/R = 0.8 kN·m^−2^.

In order to further explore the results obtained in the preceding section, we performed rheological experiments in selected PE/EVA solution mixed blends. The objective was to test the compatibility of the components in the melt state of selected systems by preparing blends that maximize the segmental interaction between the components. Melt linear rheological properties have been reported to be quite sensitive to the morphology of blends [44,45,46]. In Figure 7 (right) we observe the results obtained upon the application of the immiscible and miscible models described above to the linear viscoelastic properties G′, G″ and η″ over a wide range of frequencies (5 decades) for the blend PE_ZN/EVA 80/20 at T = 150 °C. There is excellent agreement between the experimental results and the Palierne model with a value of α/R = 0.8 kN·m^−2^. This value is in good agreement with that obtained by us in previous works [25]. It is clear, the deviation between the experiments and Palierne model from the behaviour expected for a miscible blend between the sample components. The deviation, clearly identified as an addition relaxation process at low frequencies in η″, is ascribed to the shape relaxation of the dispersed phase. This result is then in perfect agreement with that obtained from the melting point depression in Figure 5D and Figure 6, as in this case no significant changes in the melting temperature of the embedded single crystals was obtained.

The pure immiscible model does not work in the case of the presumably compatible PE0.0/EVA 80/20 blend, as observed in Figure 7 (left). The droplet shape deformation at low frequencies is expected to be more pronounced in this blend (solid lines), as a consequence of the higher difference between the rheological properties of the pure components. Notwithstanding, this sample does not fulfil the requirements of a completely miscible blend, as it does not conform with the additive rule given by Equation (3). The experimental behaviour is clearly intermediate between that expected for immiscible and miscible blends. We have applied the extension of the Palierne model developed by Lee and Denn [46]. This extension assumes that a fraction (X) of the minor component of the blend is miscible with the major component. In this case, the rheological properties of the matrix are dictated by the mixing rule in Equation (3) and the blend rheological properties are defined by the Palierne model in Equations (1) and (2), with a dispersed minor component fraction of 1 − X. This hybrid approach needs two parameters, X and α/R. α/R has been fixed at α/R = 0.8 kN·m^−2^, as in the case of a totally immiscible sample, and a suitable value of X is found to fit the experimental results. In this case, a good description of the experimental data is obtained at a value of X = 0.60. A quite similar behaviour has been found in certain blends of HDPE and LDPE [58].

**Figure 8:**
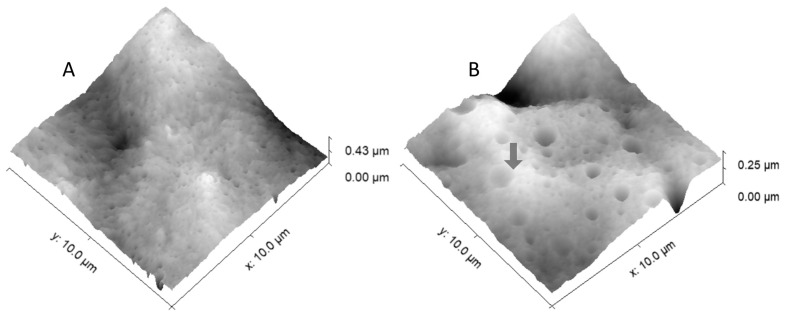
Micrographs obtained by AFM of the surface of thin films of PE0.0/EVA 80/20 blend (**A**) and PE_ZN/EVA 80/20 (**B**).

The blend morphology can be clearly observed in the AFM images in Figure 8. The semicrystalline and spherulitic nature of the PE matrices is clear in both samples, with the spherulites being bigger in PE0.0 than in ZN_PE, as expected. The amount of EVA in the blends (20 wt%) is low enough for the EVA phase to be dispersed as domains in the PE matrices. In the case of the PE0.0/EVA blend, homogeneously dispersed tiny nanodomains, around 100–200 nm are observed. On the contrary, PE_ZN/EVA, EVA domains with sizes up to 1–2 μm are clearly visible (arrow), in very good agreement with the rheological results. These sizes give interfacial tension values, α, of around 0.8–1.6 mN·m^−1^, which is in good agreement with literature results [27]. Additionally, we used the Gwyddion software to extract the mean roughness values, S_a_, from the 2D images (not shown). The evaluation of the images yields S_a_ values of 3.0 and 31.7 nm for PE0.0/EVA and PE_ZN/EVA blends, respectively.

## 4. Conclusions

The melting point depression of PE single crystals embedded in different matrices has been determined. All matrices including PCL, EAA, EVA and EMAANa cause melting depression to a certain extent but only in the single crystals obtained from the linear or slightly branched PE samples. In the case of the embedded single crystals in EVA and EMAANa matrices the depression is also much more pronounced. The single crystals obtained from the most branched and most heterogeneous PE samples do not show any sign of melting temperature depression. The study of the rheological properties confirms the trend observed in two extreme cases in which mixing at the segmental level has been promoted by solution blending. The heterogeneous PE_ZN sample is immiscible with EVA in the melt state, as probed by the Palierne model. The linear PE sample, on the other hand, fulfils a hybrid model developed for the case of partially miscible systems. The final morphology of recycled mixtures of PE (outer layer) and the materials used as tie layers studied here will depend to a great extent on the molecular architecture of the PE samples. The most branched, or more heterogeneous samples (Ziegler–Natta type), usually chosen due to the good balance of mechanical/optical properties, could be the most unsuitable, from an eco-design point of view, for recycling, as they promote immiscibility. The results show the great potential of the technique for the study of interactions between dissimilar polymers. The consequences of these interactions in terms of compatibility and recyclability are significant, and they may guide the eco-design of specific multilayer systems containing various types of polyethylene and ethylene copolymers. More research is being conducted, as it is now of interest to investigate the behaviour of common barrier polymers (such as EVOH) within this framework in order to select the most suitable (eco-designed) tie layer, promoting compatibility between the olefinic and polar layers not only during application but also during the system’s eventual mechanical recycling step.

## Figures and Tables

**Table 1:** Molecular and physical properties of polyethylene samples.

Sample	ε (%)	M_w_ (kg·mol^−1^)	M_w_/M_n_	MFI (g/10 min)	ρ (g·cm^−3^)	T_m_ (°C) ^d^
PE0.0	0.00	158	2.0	0.3	0.952	134.2
PE0.3	0.28	124	2.0	0.8	0.938	125.9
PE0.5	0.53	115	2.0	0.9	0.931	121.4
PE_ZN	1.20 ^a^/0.60 ^b^	104 ^c^	4.0 ^c^	1.0	0.918	121.0

^a^ from density measurements at T = 25 °C; ^b^ from melting temperature T_m_; ^c^ from linear rheological data and melt flow index; ^d^ from DSC experiments on pellets as received.

**Table 2:** Molecular and physical properties of copolymer samples (tie layers).

Sample	mol-% Comonomer	MFI (g/10 min)	M_w_ (kg·mol^−1^) ^d^	ρ (g·cm^−3^) ^d^	T_m_ (°C) ^e^	α ^e^
PCL	-	1.8 ^b^	50.0	1.14	58.0	0.50 ^g^
EEA	5.0%	20.0 ^c^	n.d.	0.93	54.5/94.9	0.23 ^f^
EVA	5.5%	8.0 ^c^	n.d.	0.94	52.6/84.9	0.25 ^f^
EMAANa ^a^	5.4%	4.5 ^c^	95.0	0.97	62.5/81.2	0.16 ^f^

^a^ 67 mol-% of the MAA comonomer is neutralized with Na cations [40,41]; ^b^ MFI @ 80 °C; ^c^ MFI @ 190 °C; ^d^ data from the corresponding datasheets; ^e^ from DSC experiments on pellets as received; ^f^ ΔH^0^_m_ = 293 J·g^−1^ for polyethylene [42]; ^g^ ΔH^0^_m_ = 139.5 J·g^−1^ [43].

**Table 3:** Self-seeding crystallization conditions for the preparation of single crystals (initial temperature of crystallization, T_ci_; seeding temperature, T_s_; crystallization temperature, T_c_) and thermal properties (melting temperature, T_m_; melting enthalpy, ΔH_m_; and crystallinity, α) measured by DSC.

Sample	T_ci_ (°C)	T_s_ (°C)	T_c_ (°C)	T_m_ (°C)	ΔH_m_ (J·g^−1^)	α
PE0.0	72	98	80	127.6	235.6	0.80
PE0.3	70	95	77	123.7	212.6	0.73
PE0.5	65	91	73	121.0	194.0	0.66
PE_ZN	67	93	60	122.0	170.3	0.58

## Data Availability

The data presented in this study are available on request from the corresponding authors.
